# Predicting Clinical Outcomes in COVID-19 and Pneumonia Patients: A Machine Learning Approach

**DOI:** 10.3390/v16101624

**Published:** 2024-10-17

**Authors:** Kaida Cai, Zhengyan Wang, Xiaofang Yang, Wenzhi Fu, Xin Zhao

**Affiliations:** 1 Department of Epidemiology and Biostatistics, School of Public Health, Southeast University, Nanjing 210009, China; 2 Department of Statistics and Actuarial Science, School of Mathematics, Southeast University, Nanjing 211189, China; zhengyanwang@seu.edu.cn (Z.W.); xiaofangyang@seu.edu.cn (X.Y.); 220241993@seu.edu.cn (W.F.); xinzhaomath@seu.edu.cn (X.Z.); 3 Key Laboratory of Environmental Medicine Engineering, Ministry of Education, School of Public Health, Southeast University, Nanjing 210009, China; 4 Key Laboratory of Measurement and Control of Complex Systems of Engineering, Ministry of Education, Southeast University, Nanjing 210096, China

**Keywords:** pneumonia, machine learning, missing data imputation, feature selection, COVID-19

## Abstract

In the clinical diagnosis of pneumonia, particularly during the COVID-19 pandemic, individuals who progress to a critical stage requiring mechanical ventilation are classified as mechanically ventilated critically ill patients. Accurately predicting the discharge outcomes for this specific cohort, especially those with COVID-19, is of paramount clinical importance. Missing data, a common issue in medical research, can significantly impact the validity of analyses. In this work, we address this challenge by employing two missing data imputation techniques: multiple imputation and missForest, to enhance data completeness. Additionally, we utilize the smoothly clipped absolute deviation (SCAD) penalized logistic regression method to select significant features. Our real data analysis compares the predictive performances of extreme learning machines, random forests, support vector machines, and XGBoost using 10-fold cross-validation. The results consistently show that XGBoost outperforms the other methods in predicting discharge outcomes, making it a reliable tool for clinical decision-making in the treatment of severe pneumonia, including COVID-19 cases. Within this context, the random forest imputation method generally enhances performance, underscoring its effectiveness in managing missing data compared to multiple imputation.

## 1. Introduction

Pneumonia is a prevalent infectious respiratory disease affecting individuals of all age groups globally. According to the World Health Organization, pneumonia is responsible for 75% of mortality associated with acute respiratory infections [1]. The disease can affect a wide range of individuals, including infants, people with underlying health conditions, immunocompromised individuals, the elderly, patients undergoing respiratory treatments in hospitals, those with chronic conditions such as asthma, and smokers. The severity of pneumonia varies depending on its etiology: viral pneumonia tends to be milder and may develop gradually, whereas bacterial pneumonia is often more severe and can manifest symptoms either gradually or suddenly, particularly in children [2]. Consequently, predicting the discharge outcomes for critically ill patients with suspected pneumonia holds significant clinical value. It enables healthcare professionals to manage patients more effectively and formulate personalized treatment strategies. Accurate predictions can also alert physicians to the possibility of prolonged hospital stays, allowing them to implement preventative and therapeutic interventions proactively. Moreover, this predictive insight aids healthcare facilities in optimizing resource allocation, providing critical information to patients and their families, and supporting informed decision-making.

Machine learning analyzes data by providing advanced techniques such as reinforcement learning and transfer learning algorithms, which have been successfully applied in various domains [3,4]. Currently, machine learning and artificial intelligence are widely applied in the field of healthcare. For instance, machine learning methods were used to accelerate the discovery of new functional peptides [5]. For the early diagnostic assessment of lung cancer, naive Bayes, support vector machines, decision trees, and logistic regression were employed in the predictive analysis [6]. An extreme learning machine method, based on 16 distinct features, surpassed various methodologies in forecasting postoperative survival outcomes for thoracic lung cancer [7]. The phenomenon of imbalanced data arose in predictive feature sets, for which advanced support vector machine techniques designed to tackle imbalanced data challenges were deployed to estimate postoperative life expectancy in lung cancer patients [8]. Various classification techniques, including k-nearest neighbors, artificial neural networks, decision trees, and naive Bayes algorithms were implemented, with artificial neural networks showing the most effective classification performance for breast cancer [9]. Artificial neural network technology achieved an optimal accuracy of 75.7% in predicting diabetes [10]. In the field of pneumonia research, machine learning is frequently utilized to enhance diagnostic and predictive outcomes. Stepwise multivariate binary logistic regression analyses have identified the severity of pneumonia as a predominant risk factor negatively influencing the resolution of albuminuria or hematuria and recovery from acute kidney injury (AKI) [11]. An enhanced binary Harris hawk optimization algorithm combined with a nuclear extreme learning machine offered a robust method for the accurate early assessment of COVID-19 and differentiation of disease severity [12]. To predict pneumonia in neonates, methodologies such as k-nearest neighbors, naive Bayes classifiers, decision trees, support vector machines, neural network algorithms, and random forests were applied [13]. Machine learning is also used in medical image analysis for pneumonia. For instance, the concept of transfer learning can be utilized by extracting features from images using different neural network models pre-trained on ImageNet. By combining five different models into an ensemble, state-of-the-art performance in pneumonia identification was achieved [14].

In the realm of medical research, accurately predicting patient outcomes for diseases like pneumonia, which often necessitate intensive care and mechanical ventilation, remains a daunting challenge. Traditional predictive models frequently struggle due to incomplete datasets, a common issue that potentially undermines the accuracy and usefulness of the models. Studies, such as those by Emmanuel et al. (2021), have highlighted the difficulties associated with missing data and its impact on the validity of clinical predictions [15]. Moreover, the challenge of selecting relevant features from extensive datasets without introducing bias or overfitting was well documented in the literature, with Gregorutti et al. (2017) discussing the pitfalls of including irrelevant or redundant predictors in machine learning models [16]. Our work contributes to addressing these challenges by integrating and comparing two advanced missing data imputation techniques, including multiple imputation and random forest imputation. The effectiveness of these methods in enhancing predictive accuracy in clinical settings has been supported by recent findings [17,18]. We innovate further by implementing smoothly clipped absolute deviation (SCAD) penalized logistic regression (Logit) for feature selection, a method not widely applied in medical predictive modeling but shown to significantly reduce model complexity while improving interpretability [19]. Moreover, by assessing the performance of various machine learning algorithms, including extreme learning machine, random forest, support vector machine, and extreme gradient boosting, this work expands on the work of Chen et al. (2016), who demonstrated the superior capabilities of XGBoost in handling complex, high-dimensional data [20]. This comprehensive approach not only addresses key gaps identified in the literature but also sets a new standard for predictive accuracy and practical applicability in the field of clinical decision-making.

This work aims to enhance the accuracy of discharge outcome predictions for critically ill pneumonia patients requiring mechanical ventilation by employing various machine learning models and data imputation techniques. The rest of this work is organized as follows. Section 2 outlines the missing data imputation techniques, feature selection, and machine learning methods. In Section 3, we present the outcomes of our analyses, focusing on the performance metrics of each model and the impact of the imputation methods on these metrics. Section 4 and Section 5 explore the implications of our findings, suggest future research directions for improving predictive analytics in healthcare, and highlight the key contributions of this study, emphasizing the utility of advanced machine learning techniques in clinical settings. All computations are implemented using the R programming language.

## 2. Materials and Methods

### 2.1. Data Source

In this work, we analyze a dataset from the Successful Clinical Response in Pneumonia Therapy (SCRIPT) study [21,22]. This dataset encompasses records from 12,495 patient days across 585 critically ill individuals suspected of having pneumonia and treated in intensive care units (ICUs) between June 2018 and March 2022. These patients underwent bronchoalveolar lavage as a component of routine clinical care. Each patient in the dataset is associated with binary treatment outcomes and is characterized by 48 distinct clinical features. Specifically, the dataset includes a feature “patient category”, which classifies patients into four groups: non-pneumonia control, COVID-19, other pneumonia, and other viral pneumonia. The outcome categorization defines discharges to home, acute inpatient rehabilitation (Rehab), skilled nursing facilities (SNFs), and long-term acute care hospitals (LTACHs) as favorable outcomes, assigned a value of 0. Conversely, deaths and transfers to hospice care are classified as adverse outcomes, with an assigned value of 1. In cases where patients receive lung transplantation, they are categorized under “died” to reflect the critical nature of their condition, suggesting that survival was contingent upon the transplantation. The features utilized in this analysis are comprehensively listed in Table 1, which provides the notations and interpretations for each feature. The table is formatted to facilitate straightforward reference to the specific data elements employed in our study, ensuring that the results presented are interpretable and contextualized appropriately within the clinical framework of the research.

### 2.2. Missing Data Imputation

Addressing missing data through multiple imputation is a preferred approach over single imputation, particularly because it better accounts for the uncertainty inherent in missing values. Unlike single imputation, which assigns a single estimate to each missing value and often leads to an underestimation of variance, multiple imputation creates several plausible values for each missing entry [18]. This method generates multiple imputed datasets that are then analyzed collectively to produce more accurate and statistically valid inferences. A common technique within multiple imputation is predictive mean matching (PMM). This method involves predicting the missing values by using a model based on the available data. For each missing entry, a set of similar observed values is identified, forming a donor pool. From this pool, a value is randomly selected to replace the missing data, ensuring that the imputed values are consistent with the distribution of the observed data. By repeating this process across multiple iterations, several complete datasets are generated, which together improve the reliability and robustness of the subsequent analysis.

In addition to PMM, we selected the missForest imputation method due to its nonparametric nature, which allows it to handle complex interactions between variables without making distributional assumptions [17]. This flexibility is particularly advantageous when dealing with complex clinical datasets, such as ours, where the relationships between variables may be nonlinear or difficult to model. Furthermore, missForest iteratively refines the imputed values by constructing random forests for each iteration and continues this process until the imputed values stabilize based on a convergence criterion [17]. This iterative approach ensures that the imputed values are accurate and robust, even in datasets with a variety of data types and missing patterns. The convergence is assessed by evaluating the variance or consistency of the imputed values across iterations, improving the reliability of the imputation process and making missForest a strong choice for our analysis, especially in scenarios where the missing data mechanism may not be random.

To further ensure robustness in our analysis and mitigate the sensitivity of model predictions to different types of missing data, we apply both multiple imputation and random forest imputation. Multiple imputation assumes data are missing at random (MAR), allowing for a more accurate estimation of variability and uncertainty [23,24,25]. On the other hand, random forest imputation is more flexible and effective at handling non-random missing data (MNAR), as it does not rely on distributional assumptions [17,26,27]. This combination of techniques allows us to reduce bias and improve the overall performance of our models across different missing data patterns.

### 2.3. Feature Selection Using the SCAD Penalized Method

In this study, we employ SCAD penalized logistic regression to facilitate feature selection. Logistic regression is a statistical method for analyzing a dataset in which there are one or more independent features that determine an outcome. The outcome is measured with a binary feature where there are only two possible outcomes. It is used extensively in various fields, including medicine, finance, and social sciences, for predictive analysis and inferential statistics.

The logistic regression model calculates the probability that a given input point belongs to the category labeled ‘1’, as opposed to category ‘0’. This is achieved through the logistic function, which is an S-shaped curve that can take any real-valued number and map it into a value between 0 and 1, but never exactly at those limits. The logistic function is defined as follows:

$$P\left(Y=1|X\right)=\frac{1}{1+e^{-(\beta_{0}+\beta_{1}X_{1}+\beta_{2}X_{2}+\cdots +\beta_{k}X_{p})}},$$

where P(Y=1|X) is the probability that the dependent feature *Y* equals 1 given the predictors *X*, *e* is the base of the natural logarithm, and $\beta_{0},\beta_{1},\ldots ,\beta_{p}$ are the coefficients of the model which need to be estimated from the data. These coefficients represent the change in the log odds of the outcome for a one-unit change in the predictor.

The coefficients in logistic regression are usually estimated using maximum likelihood estimation (MLE). This method seeks to find the parameter values that maximize the likelihood of the observed sample, which provides a set of equations from which the parameters can be estimated. For each observation in the dataset, the independent features are denoted $x_{i}$ and the dependent feature is denoted $y_{i}$. Let $\beta ={\left(\beta_{0},\beta_{1},\ldots ,\beta_{p}\right)}^{⊤}$, the log-likelihood function is given by the following:

$$𝓁\left(\beta \right)=\sum_{i=1}^{n}\left[y_{i}\log P\left(y_{i}=1|x_{i}\right)+\left(1-y_{i}\right)\log\left(1-P\left(y_{i}=1|x_{i}\right)\right)\right].$$

The SCAD penalty is applied to the coefficients to promote sparsity in the logistic regression model. This penalty is particularly useful for feature selection in high-dimensional data settings [19]. The SCAD penalty function is defined as follows:

$$P_{a,\lambda }\left(\beta_{j}\right)=\left\{\begin{array}{cc} \lambda |\beta_{j}| & \;\mathrm{if}\;|\beta_{j}|\leq \lambda ,\\ -\frac{|\beta_{j}{|}^{2}-2a\lambda \left|\beta_{j}\right|+\lambda^{2}}{2(a-1)}\; & \mathrm{if}\;\lambda <|\beta_{j}|\leq a\lambda ,\\ \frac{\left(a+1\right)\lambda^{2}}{2} & \;\mathrm{if}\;|\beta_{j}|>a\lambda , \end{array}\right.$$

where j=1,…,p, λ>0 and a>2 are tuning parameters. It is suggested that a=3.7 is a good choice to have satisfactory feature selection results [19]. This non-convex penalty encourages stronger shrinkage for small coefficients and less shrinkage for large coefficients, thus allowing for more effective feature selection compared to traditional methods like ridge or lasso. Feature selection and parameter estimation are concurrently achieved by maximizing the following penalized likelihood function:

$$Q\left(\beta \right)=𝓁\left(\beta \right)-\sum_{j=1}^{p}P_{a,\lambda }\left(\beta_{j}\right).$$

The application of the SCAD penalty in logistic regression not only enhances the interpretability of the model by reducing the complexity associated with less relevant features but also improves the overall predictive accuracy. This approach effectively addresses the challenges of feature selection in high-dimensional datasets, providing a robust solution for identifying significant predictors in complex models.

### 2.4. Machine Learning Methods

To identify the optimal model for predicting discharge outcomes, this work compares the performance of SCAD penalized logistic regression with that of several machine learning algorithms: extreme learning machine (ELM), random forest (RF), support vector machine (SVM), and extreme gradient boosting (XGBoost). To optimize the hyperparameters, we employ a ten-fold cross-validation approach. During this process, each fold involves using nine of the folds as the training set and the remaining one as the test set. The performance metrics from each fold are aggregated to compute an average, which informs the selection of the optimal parameters for each model. The subsequent paragraphs provide a concise introduction to these methodologies.

Extreme learning machine is a single-hidden layer feedforward neural network that differs from traditional backpropagation networks in how it initializes and trains its parameters [28]. Unlike conventional methods, ELM randomly assigns and then fixes the weights and biases between the input and hidden layers, which simplifies the training process by adjusting only the output layer weights using a least squares method. This approach has the potential to affect the prediction accuracy depending on the initialization of the hidden nodes. ELM is particularly versatile, as it supports a variety of activation functions such as Sigmoid, Tanh, and ReLU, which allow it to be adapted to different types of problems and data structures. The choice of activation function is crucial, as it determines the nonlinear transformations applied at the hidden layer, impacting the network’s overall performance.

Random forest is an ensemble learning technique used for classification and regression tasks, which consists of multiple decision trees to address the limited generalization capacity often found in individual trees [26]. It operates by constructing a multitude of decision trees, each built from random subsets of the training data and features. This randomness contributes to the model’s ability to generalize well to new data, addressing the common issue of overfitting that often plagues individual decision trees. In classification problems, random forest uses a majority voting system to decide the final class label, enhancing robustness. For instance, in binary classification, the final decision is made by selecting the class that receives the most votes across all the trees.

Support vector machine is fundamental to supervised learning, particularly in binary classification [29]. The primary goal of SVM is to identify the optimal hyperplane that effectively separates data points from two different classes. This separation is achieved by maximizing the margin, which is the distance between the hyperplane and the nearest data points from either class. The introduction of slack variables allows SVM to manage datasets that are not perfectly separable by tolerating certain misclassifications, which is particularly useful in noisy or complex data environments. The SVM algorithm’s ability to handle non-linearly separable data is enhanced by kernel functions, which map the original feature space into a higher-dimensional space where a linear separation is more feasible. This adaptability, combined with effective regularization, makes SVM a versatile and powerful classifier in a wide range of applications.

Extreme gradient boosting is an advanced ensemble machine learning method that aggregates predictions from a sequence of regression trees to compute a final score [20]. One of the key innovations in XGBoost is its use of a second-order Taylor expansion to optimize the loss function, allowing it to capture more information about the gradient and the curvature of the loss function. This leads to more accurate updates during the training process. Additionally, XGBoost includes regularization terms that penalize overly complex models, thereby improving the model’s generalization ability. The method involves iteratively adding regression trees to the model, each one correcting the errors of its predecessors, leading to a highly accurate ensemble model. XGBoost’s effectiveness in handling large-scale and high-dimensional data has made it a popular choice in various applications, including bioinformatics, where complex, high-dimensional datasets are common.

### 2.5. Performance Metrics

In this work, we evaluate the performance of classification models using three key metrics: area under the receiver operating characteristic curve (AUC), accuracy (ACC), and the F1 score. These metrics are essential to understanding different aspects of model performance comprehensively.

The AUC of the receiver operating characteristic (ROC) curve measures the overall performance of a binary classifier. The ROC curve plots the true positive rate (TPR) against the false positive rate (FPR) at various thresholds. A higher AUC value, approaching 1, indicates a better ability of the classifier to distinguish between the positive and negative classes, with a value of 0.5 representing performance no better than random chance. Accuracy quantifies the proportion of true results (both true positives and true negatives) in the total number of cases examined. It is calculated as follows:

$$\mathrm{ACC}=\frac{\left(\mathrm{TP}+\mathrm{TN}\right)}{\left(\mathrm{TP}+\mathrm{FP}+\mathrm{TN}+\mathrm{FN}\right)},$$

where TP, TN, FP, and FN represent the number of true positives, true negatives, false positives, and false negatives, respectively. The F1 score harmonizes precision and recall, which is crucial in the presence of class imbalances. Precision and recall are calculated as follows:

$$\begin{array}{cc} \mathrm{Precision} & =\frac{\mathrm{TP}}{\mathrm{TP}+\mathrm{FP}},\\ \mathrm{Recall} & =\frac{\mathrm{TP}}{\mathrm{TP}+\mathrm{FN}}. \end{array}$$

Consequently, the F1 score is derived as follows:

$$F1=2\cdot \frac{\mathrm{Precision}\cdot \mathrm{Recall}}{\mathrm{Precision}+\mathrm{Recall}},$$

offering a single metric that balances both false positives and false negatives. These metrics have been selected to ensure a robust evaluation of the models discussed in this paper, accommodating variations in class distributions and operational conditions.

## 3. Results

### 3.1. Preliminary Analysis of Missing Data and Patient Categories

**Figure 1:**
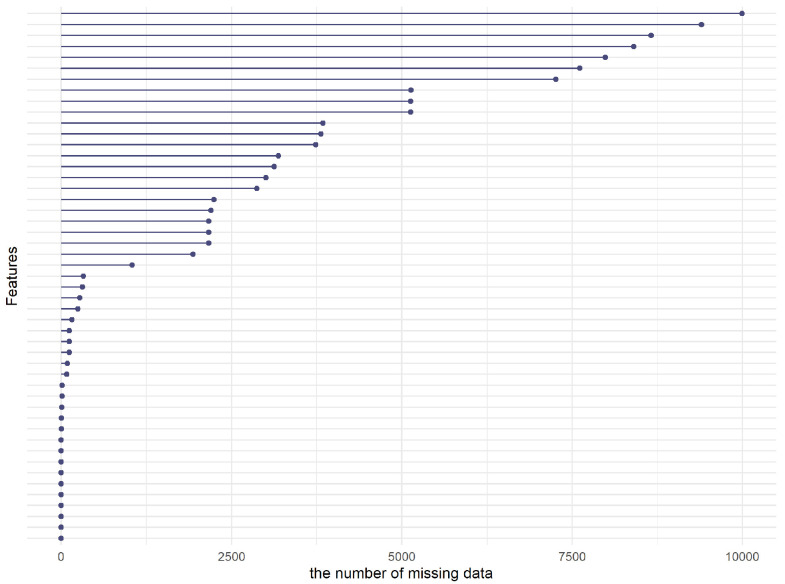
Characterization of missing features.

The missing data point numbers for all features in this work are depicted in Figure 1. Among the features analyzed, 10 exhibit missing data in excess of 40% of observations, while a significant portion, 64.6%, display missing values in fewer than 20% of cases. Specifically, 740 cases, constituting only 6% of the entire sample, have missing data exceeding 40%. To maintain the integrity and validity of our analysis, features and cases with over 40% missing data are excluded. This approach not only mitigates potential biases that could emerge from imputing substantial amounts of missing data but also streamlines the dataset, thereby enhancing the efficiency of the analysis. The refined dataset comprises 11,755 samples, incorporating 38 explanatory features and one response feature. Imputation strategies are then applied to address the remaining missing values, ensuring a robust dataset for subsequent analyses. Detailed statistics regarding missing data are provided in Table 2.

**Figure 2:**
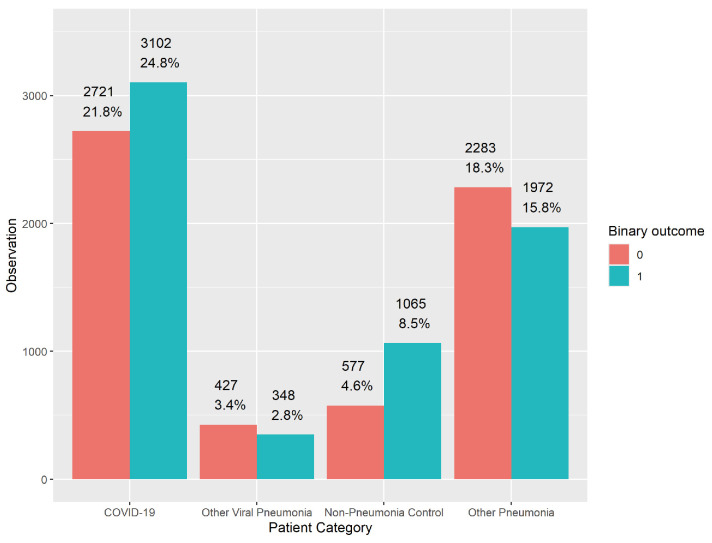
Barplots of patient categories with binary outcomes: non-pneumonia control, COVID-19, other pneumonia, and other viral pneumonia.

Given the significant impact of COVID-19 on clinical outcomes and the varying responses to treatment observed in patients with different types of pneumonia, we conducted a preliminary analysis focused on the “patient category” feature. Understanding how outcomes differ across these categories, especially in the context of the COVID-19 pandemic, is crucial for refining predictive models and enhancing clinical decision-making. The barplot in Figure 2 displays the distribution of patients across the four defined categories: non-pneumonia control, COVID-19, other pneumonia, and other viral pneumonia, with respect to the binary outcomes (0 = favorable, 1 = adverse). The visual representation indicates noticeable variations in the proportions of adverse outcomes across different patient categories. Specifically, the COVID-19 and other pneumonia groups show higher frequencies of both favorable and adverse outcomes, while the non-pneumonia control and other viral pneumonia categories have comparatively lower frequencies, with the non-pneumonia control group exhibiting a more balanced distribution between the two outcomes.

To statistically assess the association between the “patient category” feature and the binary outcome, we conduct a Chi-square test. The resulting Chi-square statistic is 182.65, with a *p*-value of less than 2.2 × 10^−16^, which is significantly below the standard significance level of 0.05. This result strongly suggests a statistically significant association between patient categories and their discharge outcomes. The evidence provided by both the barplot and the Chi-square test underlines the importance of considering patient categories in predictive modeling for discharge outcomes, particularly in critically ill patients with suspected pneumonia. This insight informs more tailored clinical decision-making processes and enhances the accuracy of outcome predictions in this patient population.

### 3.2. Identification of Important Clinical Features

Subsequent to the imputation of missing data, we employ feature selection techniques to enhance the model’s predictive performance and interpretability. Specifically, SCAD penalized logistic regression is utilized to select significant features while discarding irrelevant or redundant ones. This approach effectively mitigates the risk of overfitting and simplifies the model structure, thus facilitating clearer interpretation [19]. For the penalized logistic regression, the optimal tuning parameter values are determined through cross-validation [19]. Specifically, for the dataset imputed using multiple imputation, the tuning parameter value is 0.0067, while for the missForest imputed dataset, it is 0.0072. These optimized values ensure a balance between model complexity and predictive accuracy. In the application of SCAD penalized logistic regression, we differentiate between features selected under different imputation techniques.

The selected features, as detailed in Table 3, reflect essential clinical parameters derived from datasets processed using different imputation techniques. The patient category feature, which classifies patients into non-pneumonia control, COVID-19, other pneumonia, and other viral pneumonia, is converted into dummy features with “non-pneumonia control” as the reference category. Notably, the COVID-19 category is retained in both imputation techniques, underscoring its significant predictive value in the context of the pandemic.

Under the multiple imputation technique, the retained features span from X1 to X28, including a broad range of vital signs, laboratory results, and the COVID-19 dummy feature (X22). This extensive range suggests that multiple imputation provides a comprehensive dataset, leading to a broader base of information being maintained after feature selection. This could indicate a more conservative approach to reducing features, retaining a wide array of data points that are critical for robust model performance. Conversely, the dataset processed using the random forest imputation technique results in a similarly comprehensive selection of features, with X1 to X29 being retained, including the COVID-19 feature (X22). This selection pattern indicates that random forest imputation preserves a large number of significant predictors, including those associated with vital signs and critical biochemical markers, while still mitigating the risk of overfitting. The consistency of retaining the COVID-19 feature across both imputation methods highlights its critical importance in the predictive model, particularly in the context of viral infections.

Given the global impact of COVID-19, these results have significant implications for clinical decision-making and resource allocation, particularly in settings where COVID-19 remains a predominant concern. The inclusion of the COVID-19 feature in the final model underscores its role in predicting outcomes in critically ill patients. The ultimate effectiveness of these selected features must be evaluated in conjunction with the specific analytical methods applied subsequently, ensuring that the integration of feature selection and modeling techniques effectively addresses the clinical questions at hand.

### 3.3. Performance Evaluation of Machine Learning Methods

**Figure 3:**
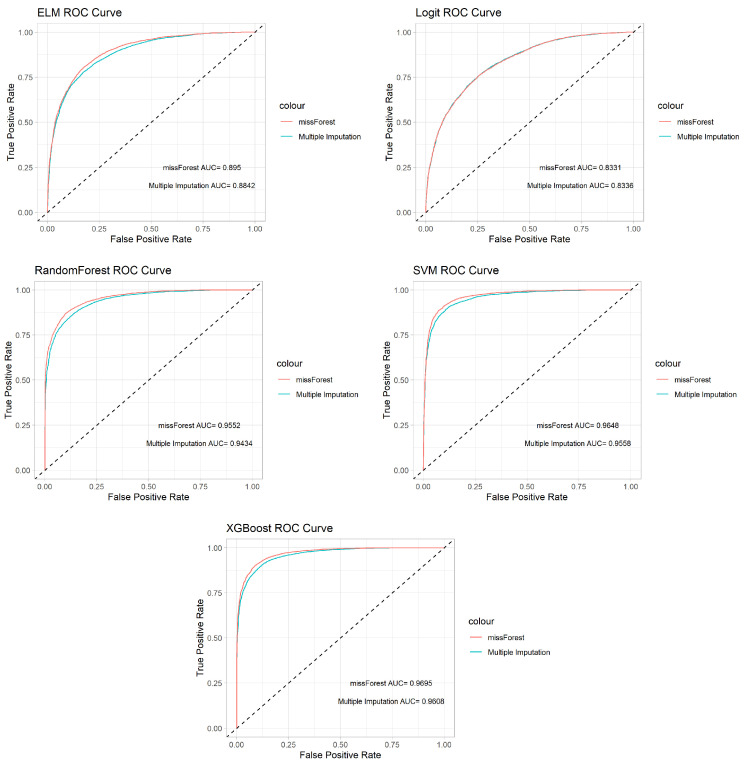
ROC plots of each analysis method with two different missing data imputation techniques.

In this study, we employ several machine learning methods to analyze real data, including extreme learning machines, random forests, support vector machines, and extreme gradient boosting. We compare the prediction performance of these methods with SCAD penalized logistic regression to identify the most effective approach. The ROC curves for each analysis method with two different missing data imputation techniques are depicted in Figure 3. This figure displays ROC curves for the same analysis method, comparing its performance across different missing data imputation techniques on a single plot. The ROC curves for each analysis method, as shown in Figure 3, indicate that the missForest imputation technique generally yields better predictive performance compared to multiple imputation across all methods. The curves for extreme learning machine, logistic regression, random forest, support vector machine, and XGBoost consistently show a slightly higher true positive rate for missForest at various false positive rates. This trend suggests that missForest more effectively captures the underlying patterns in the data, leading to improved model performance. Overall, the ROC curves demonstrate that while both imputation techniques perform similarly, missForest provides a slight but consistent advantage in predictive accuracy across different machine learning models. This enhanced performance, as illustrated by the steeper and more pronounced ROC curves, may offer critical benefits in clinical settings where even minor improvements in prediction accuracy can have significant implications for patient outcomes.

**Figure 4:**
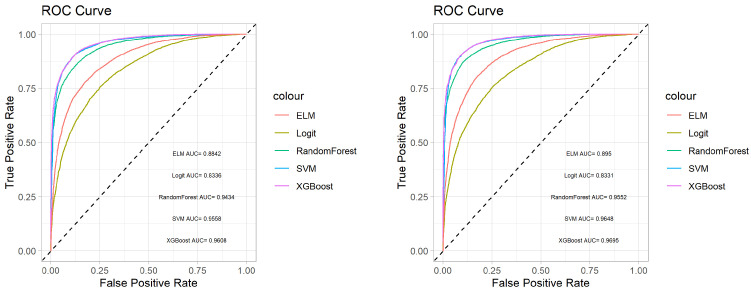
ROC plots for all analysis methods using multiple imputation on the left and random forest imputation on the right.

In Figure 4, the ROC curves of all analysis methods are plotted together to directly compare the performance of different methods under the same missing data imputation technique, highlighting the contrast between methods rather than between imputation methods. The ROC curves for each method, plotted under both multiple imputation and missForest techniques, indicate that while all models perform well, XGBoost and SVM consistently exhibit the highest predictive accuracy across both imputation methods. Notably, the missForest technique generally produces slightly better ROC curves, suggesting an enhanced ability to distinguish between outcomes. Logistic regression and ELM show comparatively lower performance, with their curves indicating less accurate predictions. The slight edge provided by missForest across methods suggests it may be the preferable imputation technique for optimizing predictive performance.

The values of performance metrics of all analysis methods for two different imputation techniques are recorded in Table 4. The performance metrics presented in Table 4 demonstrate the relative effectiveness of different machine learning methods when applied to datasets processed using two distinct imputation techniques: multiple imputation and random forest imputation. Across all methods, the random forest imputation technique consistently yields higher AUC, ACC, and F1 scores compared to multiple imputation, indicating a superior ability to handle missing data and enhance model performance. XGBoost and SVM stand out as the top-performing methods, achieving the highest AUC, ACC, and F1 scores under both imputation techniques. Specifically, XGBoost reaches an AUC of 0.9694 with random forest imputation, highlighting its robustness and suitability for high-dimensional data. Similarly, SVM shows strong performance with an AUC of 0.9649 under the same imputation method, confirming its effectiveness in classification tasks. In contrast, logistic regression consistently exhibits the lowest performance metrics among the models, particularly with only minor improvements in ACC and F1 scores when using random forest imputation. This suggests that logistic regression may be less adept at capturing complex patterns in the data compared to more advanced machine learning methods. The results indicate that random forest imputation enhances the predictive capabilities of all models, with XGBoost and SVM emerging as the most reliable choices for achieving high accuracy and robust predictions in the context of this dataset.

**Figure 5:**
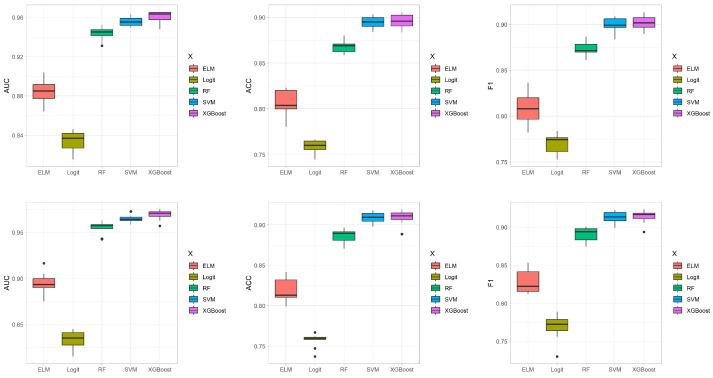
The boxplots of AUC, ACC, and F1 values for each analysis method. The three figures on the top are the results of the multiple imputation technique, and the three figures on the bottom are the results of the random forest imputation technique.

The robustness and effectiveness of XGBoost are underscored in Figure 5, where box plots display comparisons of AUC, ACC, and F1 scores obtained from ten-fold cross-validation across various models. The box plots presented in the updated figures offer a clear comparison of AUC, ACC, and F1 scores obtained from ten-fold cross-validation across various models under different imputation techniques. Across both imputation techniques, XGBoost consistently demonstrates superior performance metrics, achieving higher AUC, ACC, and F1 scores compared to other methods. Moreover, XGBoost exhibits remarkable stability across evaluations, with consistent results under both imputation techniques. In contrast, logistic regression not only shows lower overall metrics but also significant variability, especially under the random forest imputation technique, reflecting substantial fluctuations in its predictive performance. These findings highlight the robustness of XGBoost across different imputation strategies while underscoring the less stable performance of Logit, particularly when dealing with different data imputation techniques.

The confusion matrices for all analysis methods, as detailed in Table 5 and Table 6, reveal that RF, SVM, and XGBoost exhibit similar misclassification rates across various tests. However, a notable difference is observed when comparing the performance under different imputation techniques. Specifically, the use of random forest imputation significantly reduces the frequency of misclassifications, particularly in predicting adverse discharge outcomes. This improvement is evident across all models, with random forest imputation leading to fewer errors compared to multiple imputation. The substantial reduction in misclassification rates underscores the enhanced efficacy of the random forest imputation technique, particularly in boosting the predictive accuracy for critical outcomes, thereby making it a more reliable method for handling missing data in these predictive models.

In summary, the analyses conducted across various machine learning methods and imputation techniques underscore the critical role of data preprocessing in enhancing predictive performance. Among the methods evaluated, XGBoost and SVM consistently emerge as the top performers, particularly when paired with the random forest imputation technique. This combination not only yields higher AUC, ACC, and F1 scores but also demonstrates greater stability and robustness in predictive accuracy. The comparison between multiple imputation and random forest imputation techniques further highlights the latter’s superiority in reducing misclassification rates, particularly in predicting adverse outcomes. These findings suggest that employing advanced machine learning models like XGBoost, along with effective imputation strategies such as random forest imputation, can significantly improve the reliability of predictions in clinical settings, ultimately contributing to better patient outcomes.

## 4. Discussion

The findings from this study provide compelling evidence supporting the integration of advanced machine learning methods and sophisticated imputation techniques to enhance the predictive accuracy of clinical outcomes in critically ill pneumonia patients. The consistent superiority of methods like XGBoost and SVM across various performance metrics underscores the importance of using state-of-the-art machine learning approaches in medical decision-making processes. These methods, particularly when combined with the random forest imputation technique, demonstrated robust performance in predicting discharge outcomes, achieving higher AUC, ACC, and F1 scores compared to traditional methods such as logistic regression.

One of the key observations from our study is the impact of the chosen imputation technique on the predictive performance of the methods. Random forest imputation consistently outperformed multiple imputation, as evidenced by the higher true positive rates and reduced misclassification errors across different methods. This suggests that random forest imputation more effectively handles the complexities and nuances inherent in the dataset, particularly in capturing the underlying patterns that contribute to accurate predictions. This finding aligns with previous research that highlights the efficacy of nonparametric imputation methods in dealing with missing data in high-dimensional settings.

The selection of significant features using SCAD penalized logistic regression further enhanced the interpretability of the methods without compromising their predictive power. The retention of the COVID-19 feature across different imputation methods, for instance, highlights its critical role in predicting patient outcomes during the pandemic. This insight is particularly relevant in the current global healthcare context, where COVID-19 remains a predominant concern, influencing clinical decisions and resource allocation. The “patient category" feature, particularly in the context of COVID-19, plays a critical role in enhancing prediction accuracy. COVID-19 patients tend to exhibit distinct clinical characteristics, such as higher rates of comorbidities, systemic inflammation, and atypical respiratory failure, compared to other pneumonia patients [30,31,32]. By capturing these unique aspects, the model can improve its prediction of patient outcomes, including ICU admissions, the need for mechanical ventilation, and length of hospital stay. The SCAD penalty’s ability to promote sparsity while retaining significant predictors ensures that the methods remain both interpretable and effective, which is crucial for their application in clinical settings.

Additionally, it is important to highlight the practical implications of using different machine learning methods for clinical decision-making. Methods like random forest and XGBoost, which are capable of handling complex interactions between features, are particularly well-suited for clinical environments where patient data are often heterogeneous and nonlinear relationships are common. Their ability to deliver robust predictions, even with missing data, makes them highly suitable for real-time decision-making in critical care settings. On the other hand, SCAD penalized logistic regression offers interpretability, which is critical for clinicians who need to understand the key features driving the predictions. By using a combination of these methods, healthcare providers cannot only enhance prediction accuracy but also ensure transparency and trust in model-driven decisions.

Despite these promising results, it is important to acknowledge the limitations of this study. The dataset, while comprehensive, is specific to a particular population and time period, which may limit the generalizability of the findings to other settings or patient groups. Furthermore, while the machine learning methods employed in this study showed high accuracy, their implementation in real-world clinical settings requires careful consideration of factors such as method interpretability, ease of integration with existing systems, and the potential for over-reliance on automated decision-making tools. Future research should explore the applicability of these methods across diverse populations and investigate the integration of these tools into clinical workflows to ensure they complement rather than replace clinician judgment.

Overall, this study contributes to the growing body of evidence supporting the use of advanced machine learning techniques in healthcare. By demonstrating the effectiveness of methods like XGBoost and SVM in combination with random forest imputation, this work lays the groundwork for future studies aimed at refining these methods and exploring their broader applicability in clinical practice.

## 5. Conclusions

In conclusion, this study demonstrates the effectiveness of advanced machine learning methods and data imputation techniques in predicting discharge outcomes for critically ill patients with pneumonia. Among the methods evaluated, XGBoost and SVM consistently outperformed other methods, particularly when combined with random forest imputation. This combination not only improved predictive accuracy but also reduced misclassification rates, highlighting its potential utility in clinical decision-making.

This study underscores the critical role of data preprocessing, particularly in handling missing data, in enhancing the performance of the predictive method. The findings suggest that employing robust imputation techniques, such as random forest imputation, can significantly improve the reliability of machine learning methods in medical research. Furthermore, the inclusion of the “patient category” feature, particularly in the context of COVID-19, has proven to be a vital factor in improving the accuracy of outcome predictions. The results of this study provide a strong foundation for further research and the development of more refined predictive methods, which could be invaluable in clinical settings where timely and accurate predictions are crucial for patient care.

The methodologies and findings presented in this study have important implications for the development of predictive analytics in healthcare. By leveraging advanced machine learning techniques and rigorous data preprocessing methods, healthcare professionals can enhance the accuracy of their predictions, ultimately leading to better patient outcomes and more efficient use of healthcare resources. Future research should continue to explore the integration of novel machine learning approaches and imputation techniques to further refine and optimize predictive methods for various clinical applications.

## Figures and Tables

**Table 1:** The notations and interpretations of features.

Feature	Interpretations
Y	Binary indicator of patient outcome for this admission (0 = good outcome, 1 = poor outcome).
X1	Binary indicator of whether the patient was on ECMO on this day (0 = No, 1 = Yes).
X2	Binary indicator of whether the patient received dialysis on this day (0 = No, 1 = Yes).
X3	Identifier for which ICU stay this is if a patient has multiple stays during one hospitalization.
X4	Day number of the current ICU stay (starting from 1 at 12:00 a.m. on the first day).
X5	Sequential Organ Failure Assessment (SOFA) score for the day.
X6	Average body temperature in degrees Fahrenheit.
X7	Average heart rate in beats per minute.
X8	Average systolic blood pressure in mmHg.
X9	Average diastolic blood pressure in mmHg.
X10	Average respiratory rate per minute.
X11	Average oxygen saturation percentage.
X12	Lowest Glasgow Coma Scale motor response score for the day.
X13	Median Richmond Agitation Sedation Scale score for the day.
X14	Average positive end-expiratory pressure (PEEP) in cm H_2_O.
X15	Average fraction of inspired oxygen percentage.
X16	Number of changes to PEEP settings on the ventilator throughout the day.
X17	Number of changes to the respiratory rate settings on the ventilator throughout the day.
X18	Number of changes to FiO_2_ settings on the ventilator throughout the day.
X19	Worst PaO_2_/FiO_2_ ratio recorded during the day.
X20	Average white blood cell count in K/UL.
X21	Average absolute lymphocyte count in K/UL.
X22	Patient category, including non-pneumonia control, COVID-19, other pneumonia and other viral pneumonia.
X23	Average hemoglobin level in g/dL.
X24	Lowest platelet count in K/UL for the day.
X25	Average bicarbonate level in mmol/L.
X26	Average albumin level in g/dL.
X27	Highest bilirubin level (part of SOFA score) in mg/dL.
X28	Binary indicator of whether the patient was intubated on this day (0 = No, 1 = Yes).
X29	Highest creatinine level in mg/dL recorded during the day.

**Table 2:** The percentage of missing data points for features and samples.

Percentage of Missing Data	Features		Sample	
	Frequency	Percentage (%)	Frequency	Percentage (%)
[0, 0.2]	31	64.6	7238	57.8
(0.2, 0.4]	7	14.6	4517	36.2
(0.4, 0.6]	4	8.3	719	5.8
(0.6, 0.8]	6	12.5	21	0.2
(0.8, 1]	0	0	0	0

**Table 3:** Selected features of SCAD penalized logistic regression under different imputation techniques.

Imputation Technique	Selected Features
Multiple imputation	X1, X2, X3, X4, X5, X6, X7, X8, X9, X10, X11
	X12, X13, X14, X15, X16, X17, X18, X20, X21
	X22, X23, X24, X25, X26, X27, X28
Random forest imputation	X1, X2, X3, X4, X5, X6, X7, X8, X9, X10, X11
	X12, X13, X14, X15, X16, X17, X18, X19, X20
	X21, X22, X23, X24, X26, X27, X28, X29

**Table 4:** Performance metrics of all analysis methods for two different imputation techniques.

Model	Multiple Imputation			Random Forest Imputation		
	AUC	ACC	F1	AUC	ACC	F1
Extreme Learning Machine	0.8844	0.8062	0.8087	0.8949	0.8191	0.8287
Logistic Regression	0.8338	0.7591	0.7702	0.8332	0.7571	0.7691
Random Forest	0.9433	0.8680	0.8732	0.9551	0.8863	0.8908
Support Vector Machine	0.9558	0.8948	0.8993	0.9649	0.9087	0.9130
XGBoost	0.9606	0.8960	0.9023	0.9694	0.9092	0.9139

**Table 5:** Confusion matrix of all analysis methods for the multiple imputation technique.

Multiple Imputation	ELM		Logit		RF		SVM		XGBoost	
	0	1	0	1	0	1	0	1	0	1
0	4649	868	4171	1346	4854	663	4987	530	4884	633
1	1410	4828	1486	4752	889	5349	707	5531	590	5648

**Table 6:** Confusion matrix of all analysis methods for the random forest imputation technique.

missForest	ELM		Logit		RF		SVM		XGBoost	
	0	1	0	1	0	1	0	1	0	1
0	4479	1038	4261	1256	4886	631	4980	537	4964	553
1	1200	5038	1620	4618	760	5478	707	5531	604	5634

## Data Availability

The data presented in this study are available on PhysioNet at https://doi.org/10.13026/5phr-4r89.

## Abbreviations

The following abbreviations are used in this manuscript:

SCAD	smoothly clipped absolute deviation
Logit	logistic regression
RF	random forest
SVM	support vector machine
XGBoost	extreme gradient boosting
ELM	extreme learning machine
ROC	receiver operating characteristic
AUC	area under the curve
ACC	accuracy
COVID-19	Coronavirus Disease 2019
